# A scoping review of regenerative medicine in medical education

**DOI:** 10.1186/s12909-022-03816-7

**Published:** 2022-11-05

**Authors:** Rachita Pandya, Zohray Talib

**Affiliations:** California University of Science and Medicine, 1501 Violet St, Colton, CA 92324 USA

**Keywords:** Medical education, Regenerative medicine, Stem cell therapeutics, Clinician training

## Abstract

Stem cell therapeutics and regenerative medicine have taken a strong foothold in biomedicine. However, most physicians are currently not adequately prepared to identify, refer, and deliver safe regenerative therapies. To understand this gap, we sought to characterize published literature on current physician training in regenerative medicine. Our scoping review describes current training strategies to bridge the gap and integrate such education into medical curricula for adequate training.

**Supplementary Information:**

The online version contains supplementary material available at 10.1186/s12909-022-03816-7.

## Background

The goal of medical education is to produce clinicians who provide consistent and high quality care while also serving as transformative change agents efficiently and effectively stewarding resources in the health care system [1]. Achieving this ambitious goal will require a workforce that has foundational knowledge of medicine yet is equipped to learn and use new technologies as they are developed. The Lancet Commissions for Education of Health Professionals laid out the evolution of medical education from informative to formative to transformative [1]. Informative learning is focused on the acquisition of skills and knowledge and produces experts [1]. Formative learning aspires to produce a workforce that has expertise and overlay a set of values, resulting in a cadre of professionals [1]. Transformative learning aims to produce transformative change agents who can leverage their expertise, apply a set of values and navigate the broader system to produce change [1]. Transformative change agents can then leverage new technologies for the right patient at the right time [1]. The current field of regenerative medicine presents an opportunity for medical education to actualize the vision of the Lancet Commission. A robust health workforce for the twenty-first century would include practitioners who are versed and ready to use new regenerative medicine technologies.

Regenerative medicine is one such technology that has the potential to change the way clinical medicine is practiced. Despite the incredible advances in regenerative medicine and their relevance across the non-communicable diseases which are impacting health outcomes globally, the implementation of regenerative medicine into clinical care has been slow [2]. To date, several barriers have been identified that explain the limited physician engagement seen with regenerative medicine. These include the stigma that cell therapy is based on embryonic cell lines, a lack of adequate formal training, and a lack of adequate basic science knowledge to navigate the field. Widespread integration of regenerative medicine topics and training into medical education curricula requires an understanding of the lessons learned from institutions that have taken this step. Accordingly, the objectives of this scoping review are to characterize the published literature and conduct a thematic analysis on current physician training in regenerative medicine.

## Methods

As the aim of our study was to characterize the literature in the emerging field of regenerative medicine and medical education, with a focus on analyzing the current body of published literature, we selected a scoping review as the best approach. We conducted our scoping review following the Joanna Briggs Institute guidelines [3].

### Database sources and search strategy

RP searched three electronic academic databases (PubMed, ScienceDirect, and SCOPUS) for literature published between January 2010 and May 2022. We decided to focus on these three databases due to their scope and relevance to the fields of education and science. An initial screening of publications in the field of regenerative medicine showed an uptrend from 2010 onwards which informed our timeline. We included journal articles and review papers. We did not consider abstracts and conference proceedings as they were unlikely to provide sufficient detail on regenerative medicine curricular models. No language limitations were used. The search field was a string of two keywords agreed upon by the authors to capture stem cell therapeutics and physician training. Keywords for stem cell therapeutics included ‘regenerative medicine’ and ‘stem cell research’. Keywords for education included ‘medical education’, ‘physician education’, and ‘clinician education’. This created the following search strings used for each database: "regenerative medicine" and "medical education", "regenerative medicine" and "physician education", "regenerative medicine" and "clinician education", "stem cell research" and "medical education", "stem cell research" and "physician education", and "stem cell research" and "clinician education" (please see Additional file 1 for further details). All citations were imported into the web-based reference management software Mendeley 1.19.4 (Mendeley, London, UK). Duplicate entries present in two or more databases were identified digitally and removed. Subsequent screening of titles and abstracts was also conducted within this software.

### Screening criteria

A two-step eligibility process was used. Articles met inclusion criteria only if they 1) were related to the field of regenerative medicine 2) discussed a training program 3) if the trainees were pre-medical students, medical students or physicians. Articles that were excluded were those that reflected on regenerative medicine without describing training needs or training programs for clinicians. For the first step of screening, RP and ST independently reviewed the titles and abstracts to assess if they met the inclusion criteria. Prior to starting independent screening on the full dataset, the two reviewers conjointly screened a small subset of studies. For pertinent titles whose abstracts were unavailable, full article review was conducted in the subsequent screening phase. Reviewers convened again at the end of the screening stage to discuss uncertainties in study inclusion and resolve any differences.

### Data analysis

Following the screening process, full text content analysis was conducted for all articles that met inclusion criteria by RP and ZT. A deductive approach was used initially to categorize the topics, types of trainees and desired outcomes. An inductive approach was then used to identify themes related to opportunities, challenges and lessons learned. Sheets spreadsheet (Google, LLC, Mountain View, CA) were used to track the analysis.

## Results

**Fig. 1:**
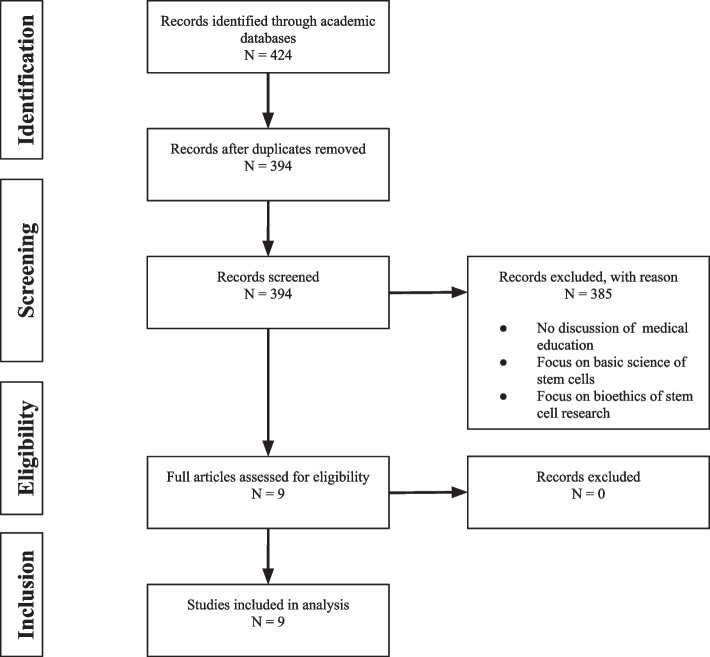
PRISMA flow diagram for scoping review schema

Searches of the three academic databases yielded a total of 424 citations. Following deduplication, 394 citations remained. After review of the titles and abstracts in the first screening level, 385 citations were excluded due to lack of discussion on clinician training. The 9 remaining citations were confirmed to meet inclusion criteria through full-text analysis and referenced training for physicians in regenerative medicine. A PRISMA flow diagram of the identification and selection process for articles is depicted in Fig. 1 [4, 5].

All included studies were published between 2011 and 2021, and all were journal articles. Five of the articles discussed existing training programs and one discussed a proposed training program. Most publications included first authors from within the US. Two are from Florida, two are from California, and one is from Minnesota. Four publications have international first authors. One is from Canada, one is from China, one is from Germany, and one is from Iran.

### Types of programs

We identified five programs that incorporated regenerative medicine into medical education and one proposed program (Tables 1 and 2). These programs included some that offer exposure to future physician-scientists, and others that offered trainees the opportunity to develop proficiency in clinical and surgical skills needed to practice. The trainees in the programs were diverse. Two programs included medical students, two programs included graduate medical residents, four targeted fellows, and two targeted graduate students. Two programs included undergraduate students or professionals from other disciplines, like engineering and biology.

**Table 1:** Summary of existing training programs

Program	Trainees	Learning Model	Expected Outcomes	Measures of Success
CRISDP at University of Florida Regional Center [6]	Cardiovascular medicine, cardiothoracic surgery, and other cardiovascular medicine-related fellows	Course series on basic science of cell therapy and mentored research participation	Equip future physician-scientists with skills for cardiovascular regenerative medicine	Completion of coursework, presentations at meetings, publication, and academia positions
Regenerative Medicine and Surgery Course at Mayo Clinic [7]	Undergraduate medical students, graduate students, internal medicine residents, and research fellows	Didactic lectures on regenerative medicine principles, laboratory experience with regenerative technology, and surgical practice on cadavers	Provide training in regenerative models for clinical solutions	Examinations and research advancement
The Berlin-Brandenburg School for Regenerative Therapies [8]	Medical students, clinical scientists, biologists, and engineers	Incubator model; active research participation and clinical rotations for needs assessment	Providing interdisciplinary training to promote translational research and clinical application	Defense of research thesis
Cardiovascular Cell Therapy Research Network at NHLBI [9]	Medical students and residents	Basic science research, imaging modality training, cardiovascular assessment in animal models, and courses on how to carry out clinical trials	Provide future physician-scientists with expertise in regenerative medicine practices related to cardiovascular diseases	Research progress and completion
Canadian Stem Cell Network [10]	Undergraduate/graduate students and postdoctoral fellows	Seminars, workshops, committee memberships, and networking opportunities	N/A	N/A

**Table 2:** Description of training program models

The Clinical Research Investigator Skills Development Program at the University of Florida Regional Center (CRISDP–UFRC) [6]	The Clinical Research Investigator Skills Development Program at the University of Florida Regional Center (CRISDP–UFRC) provides trainees with the opportunity to gain skills in cardiovascular regenerative medicine [6]. The training program is open to cardiovascular medicine and surgery fellows, and those from other cardiovascular-related disciplines [6]. This program employs both a didactic and mentored laboratory experience as a part of its learning model [6]. Trainees are enrolled in a course series focusing on the basic sciences of stem cells and regenerative medicine, taught by departmental faculty [6]. This is coupled with practical training under the guidance of technical mentors [6]. Eligible mentors are expert scientists with a background in cardiac stem cell, stem cell precursors, mesenchymal stem cell progenitors, and cell delivery biology [6]. Trainees are assessed on adequate completion of coursework, documented scientific progress, and future acquisition of academic positions [6]
The Regenerative Medicine and Surgery Course at Mayo Clinic [7]	The Regenerative Medicine and Surgery Course at Mayo Clinic was created with the goal of increasing student literacy of and interest in regenerative medicine concepts [7]. Participants in the program include 1st, 2nd, and 4th year medical students, graduate students (Ph.D and MD/Ph.D), PGY-1 and 2 internal medicine residents, and research fellows [11]. Trainees participate in a series of didactic lectures focused on regenerative medicine principles, daily briefings on clinical trials to understand regenerative approaches within the clinic, laboratory experiences in stem cell culturing and 3D bioprinting, cadaver demonstrations of surgical procedures, and a regenerative medicine career panel [7]. The laboratory demonstrations are conducted by a multidisciplinary team of faculty members from sports medicine, cardiology, plastic surgery, orthopedic surgery, and otolaryngology [7]. To ensure trainees are meeting the learning objectives, assessment is conducted via online tests and simulated clinical exams [7]
The Interdisciplinary Stem Cell Institute (ISCI) at the University of Miami Medical School through NHLBI [9]	The Interdisciplinary Stem Cell Institute (ISCI) at the University of Miami Medical School trains fellows with the goal of developing a skill set in stem cells and regenerative pathway research techniques [9]. One component of the training program is a basic science research experience, under the mentorship of ISCI faculty, to learn stem cell culture and molecular biology techniques along with good manufacturing practice for stem cells [9]. The Department of Radiology faculty provide training in imaging modality training, including acquisition and analysis [9]. Also, trainees obtain extensive training from interventional cardiologists on the delivery and assessment of molecular therapies in animal models monitored for cardiovascular dynamics [9]. Lastly, trainees complete courses on how to conduct clinical trials [9]. Trainee assessment occurs through team discussions, laboratory meetings and presentations, and study progress [9]
The Berlin-Brandenburg School for Regenerative Therapies (BSRT) [8]	The Berlin-Brandenburg School for Regenerative Therapies (BSRT) utilizes a three-pronged approach to train its scholars [8]. Medical students are eligible for the junior clinical scientist program that spans the four years of their undergraduate medical education [8]. Students participate in summer courses and research preparation for the nine-month dedicated research term that follows, during their studies [8]. Exemplary completion of this program provides the opportunity for further training through the clinical scientist program [8]. In the clinical scientist program, future clinical scientists are enrolled in the PhD program and receive funding to start their own research [8]. Lastly, for the PhD biology and engineering students, BSRT offers clinical exposure in both a hospital and outpatient setting, for a needs assessment and opportunity to understand the application of their studies [8]
The Canadian Stem Cell Network (SCN) [10]	The Canadian Stem Cell Network (SCN) was established in 2001 and promotes stem cell research, with the goal of training future stem cell scientists [10]. Trainees within this network can include undergraduate and graduate students, from both medical and non-medical backgrounds, research associates, and postdoctoral fellows [10]. The network provides its trainees with a variety of opportunities to garner knowledge and skills within the field: workshops, seminars, leadership opportunities within committees, a platform to network with other professionals, and education on policy and ethics surrounding stem cells [10]. A 2015 focus group study of graduate students and postdoctoral fellows conducted on SCN trainees indicated that a majority remained in an academic setting, while fewer moved to the industry, private, or government sectors [10]
*Proposed Model* [12]	The proposed model suggests a one-year fellowship in stem cell-based regenerative medicine that can follow residency programs, like internal medicine, surgery, or hematology/oncology [12]. Trainees would receive education on ethics, regulations, basics of cell biology, and stem cell-specific techniques [12]. With hematology/oncology being the only current field qualified in delivering hematopoietic stem cell therapies, such a fellowship program would be established in conjunction with an institution’s internal medicine department and taught by hematology/oncology faculty [12]. Ultimately, successful completion of this program would lead to certification in “regenerative and cellular medicine” [12]

The content of the programs ranged from basic science principles on regenerative medicine to coursework on how to conduct clinical trials. One of the programs included the opportunity to develop procedural skills. In three of the programs, trainees were engaged in an actual research project. Two of the programs offered trainees the opportunity to identify mentors and gain exposure to a network of experts in the field.

The pedagogy to deliver these curricula included didactic sessions, wet laboratory work, cadaver lab experiences, seminars, and networking events. Three of the programs were solely medical education programs, whereas three were interdisciplinary (including learners from different health science programs). Four out of the five programs assessed their trainees through research work, two programs assessed using an examination, and one program did not have an assessment.

### Measures of success

Each of the existing training programs utilized various methods to gauge learner progress and completion, and efficacy of the training program (Table 1). All programs used research project advancement and completion, one program also used coursework completion, one program also used number of publications, and one program also used post-graduation academia positions.

### Additional thematic analysis

In addition to the types and nature of training programs, three additional themes emerged from a content analysis of the papers reviewed in this scoping review; All the papers reflected on the critical and time-sensitive need for a trained workforce; The lack of professional standards or curricula for training a workforce; The critical impact of this deficit on patient safety.

### *Training as a bottleneck to scaling the use of new technologies in clinical care*

Studies reported that while there has been funding and efforts in the biomedical science to demonstrate the safety and clinical outcomes of cell-based therapies, there appears to be lag in the translation of these technologies into clinical care [2, 6, 8]. This inability to translate basic science discoveries into clinical application is in part, attributed to the lack of health professionals with the knowledge and clinical skills proficiencies to provide safe and effective care [8, 11].

### *Lack of standards of training*

The reports noted that regenerative medicine topics are not currently required, and no accepted curriculum exists for medical education [7]. Without an official training or board certification process, a formal workforce of practitioners and surgeons does not exist to facilitate cell therapies. The lack of basic knowledge of stem cell therapeutics among practitioners also stifles their ability to investigate or participate in research around these therapeutics [2, 11, 13].

### *Lack of standards and guidelines for clinical care*

It was noted in the papers published that in the health care service setting, stem cell therapies are so new that there is no clarity on where one might receive these treatment options and which health care professionals would offer or discuss this. This lack of clarity on the specific roles and responsibilities of the health workforce as it relates to stem cell therapy, makes it difficult then for patients to initiate the discussion, not knowing who to turn to [13]. One paper also noted that the lack of current regulations and standards has allowed untrained professionals to offer these treatments, potentially bringing into question patient safety concerns [9].

### *Urgency of developing standards for medical education for patient safety*

Almost all the published reports noted an urgent need for the development of formal training programs in regenerative and cellular medicine [2, 7–9, 11, 12]. They noted that clinicians would require foundational knowledge and clinical skills training to ensure the provision of safe cell therapy in a clinical setting [2, 7–9, 12]. Ideally, these topics would be introduced early in training to allow for progressive development of skills and expertise [6].

## Discussion

This study is the first scoping review published to characterize the literature and understand the state of regenerative medicine training models in medical education curricula.

Our study revealed that of the five currently identified training programs, all are offering similar breadth in terms of first a foundational education on stem cell basic science knowledge and then opportunities to obtain relevant technical and clinical skills. Interestingly, the focus of these training programs is to produce clinician scientists who can continue to advance regenerative medicine as an emerging field. Ultimately, if regenerative medicine fulfills its promise of wide application, there will likely be a need for clinicians who use these skills and therapies in routine clinical practice. The broad scope of regenerative medicine, offering treatment options across the different organ systems, means that physicians of all disciplines should ideally be well-versed. The future workforce, therefore, would not be limited to those in a clinical research track. In fact, if regenerative medicine becomes standard of care, all physicians should at least be fluent in the basic knowledge of regenerative medicine to be able to identify potential patients and to provide basic patient education.

One of our observations in this review is that existing training programs are being offered at a variety of stages during medical education, which makes it difficult to create a standardized curriculum. The next leap forward in this field would be a foundational curriculum that covers the basic concepts of regenerative medicine, along with a curated set of core regenerative medicine competencies that physicians, of all disciplines, should meet to safely and appropriately deliver, educate or counsel on related to regenerative medicine.

The training programs reviewed in this study reinforce the vision that regenerative medicine should ideally be taught across different phases of medical education including pre-clinical, clinical, and even post-graduate education. Training should ideally include activities that provide knowledge as well as the opportunity to develop clinical skills alongside longitudinal mentorship. Rather than all schools re-inventing similar curricula, it would be a leap forward and a likely a catalyst for the workforce if an open-source, best-practice, foundational curriculum were developed that all schools could leverage in their undergraduate curricula. This could ensure that all students the basic knowledge of the science, the utility and the opportunities of regenerative medicine. Programs could then develop opportunities for advanced training for those who intend to engage in clinical practice of regenerative medicine. Fellowships in a range of specialties are likely to emerge as validated, approved therapies emerge to treat cardiac conditions, orthopedic conditions, dermatology conditions etc.

This scoping review presents with limitations. In spite of a board search strategy across three major academic databases, this review reflects only those training programs that have published on their programs. It is likely that many more programs exist but remain unpublished. This may likely have led to a publication bias. We surmise that training programs at larger and well-funded medical institutions may have greater capacity for and inclination to publish about their education research. Additionally, the thematic extractions in this study are based upon a sample size of *N* = 9 and may not be representative of best practices for integrating regenerative medicine into medical education curricula. Lastly, during the time that we conducted our study, PubMed underwent a change in its architecture, which has altered its filter categories (https://www.nlm.nih.gov/pubs/techbull/so22/so22_issue_cover.html). This may affect the number of results produced with each search string when trying to re-run the search in the current time.

### Future perspective

The discovery of stem cell therapeutics has outpaced physician training. However, as the landscape of patient care and needs shift towards restoration of physiology with the advent of regenerative medicine, physicians in all specialties will require at least basic training in regenerative medicine to ensure the safe and appropriate use of these novel and highly impactful therapies. Implementation of regenerative medicine topics throughout the medical education curricula, using a phased, competency-based approach, will be critical to generating a health workforce that can recognize and practice safe stem cell therapeutics.

## Supplementary Information

**Additional file 1: Table 1.** Search strategy details for ScienceDirect. **Table 2.** Search strategy details for SCOPUS. **Table 3.** Search strategy details for PubMed

## Data Availability

All data generated or analyzed during this study are included in this published article.
