# Supplementary material for: A scoping review of regenerative medicine in medical education
Source: BMC Med Educ. 2022 Nov 5;22:758. doi: 10.1186/s12909-022-03816-7 (PMC9636647; doi:10.1186/s12909-022-03816-7)
Supplement: Supplementary file 1 — Additional file 1: Table 1. Search strategy details for ScienceDirect. Table 2. Search strategy details for SCOPUS. Table 3. Search strategy details for PubMed [file 12909_2022_3816_MOESM1_ESM.docx]

Table 1. Search strategy details for ScienceDirect

| **Search String for ScienceDirect** | **Search Conducted** | **Years of Search** | **Article Types** | **# of Results** |
| --- | --- | --- | --- | --- |
| **“regenerative medicine” and “medical education”** | Search 1  Nov 13, 2020  Search 2  May 25, 2022 | Search 1  2010-2020  Search 2  2021-2022 | Search 1  review and research articles  Search 2  review and research articles | Search 1  112 (<https://www.sciencedirect.com/search?qs=%E2%80%9Cregenerative%20medicine%E2%80%9D%20and%20%E2%80%9Cmedical%20education%E2%80%9D&date=2010-2020&articleTypes=REV%2CFLA>)  Search 2  41 |
| **“stem cell research” and “medical education”** | Search 1  Nov 13, 2020  Search 2  May 25, 2022 | Search 1  2010-2020  Search 2  2021-2022 | Search 1  review and research articles  Search 2  review and research articles | Search 1  33  Search 2  7 |
| **“regenerative medicine” and “physician education”** | Search 1  Nov 13, 2020  Search 2  May 25, 2022 | Search 1  2010-2020  Search 2  2021-2022 | Search 1  review and research articles  Search 2  review and research articles | Search 1  1  Search 2  3 |
| **“stem cell research” and “physician education”** | Search 1  Nov 13, 2020  Search 2  May 25, 2022 | Search 1  2010-2020  Search 2  2021-2022 | Search 1  review and research articles  Search 2  review and research articles | Search 1  0  Search 2  2 |
| **“regenerative medicine” and “clinician education”** | Search 1  Nov 13, 2020  Search 2  May 25, 2022 | Search 1  2010-2020  Search 2  2021-2022 | Search 1  review and research articles  Search 2  review and research articles | Search 1  0  Search 2  0 |
| **“stem cell research” and “clinician education”** | Search 1  Nov 13, 2020  Search 2  May 25, 2022 | Search 1  2010-2020  Search 2  2021-2022 | Search 1  review and research articles  Search 2  review and research articles | Search 1  0  Search 2  0 |

Table 2. Search strategy details for SCOPUS

| **Search String for SCOPUS** | **Search Conducted** | **Years of Search** | **Article Types** | **# of Results** |
| --- | --- | --- | --- | --- |
| **“regenerative medicine” and “medical education”** | Search 1  Nov 13, 2020  Search 2  May 25, 2022 | Search 1  2010-2020  Search 2  2021-2022 | Search 1  article and review  Search 2  article and review | Search 1  54  Search 2  14 |
| **“stem cell research” and “medical education”** | Search 1  Nov 13, 2020  Search 2  May 25, 2022 | Search 1  2010-2020  Search 2  2021-2022 | Search 1  article and review  Search 2  article and review | Search 1  25  Search 2  2 |
| **“regenerative medicine” and “physician education”** | Search 1  Nov 13, 2020  Search 2  May 25, 2022 | Search 1  2010-2020  Search 2  2021-2022 | Search 1  article and review  Search 2  article and review | Search 1  3  Search 2  1 |
| **“stem cell research” and “physician education”** | Search 1  Nov 13, 2020  Search 2  May 25, 2022 | Search 1  2010-2020  Search 2  2021-2022 | Search 1  article and review  Search 2  article and review | Search 1  0  Search 2  1 |
| **“regenerative medicine” and “clinician education”** | Search 1  Nov 13, 2020  Search 2  May 25, 2022 | Search 1  2010-2020  Search 2  2021-2022 | Search 1  article and review  Search 2  article and review | Search 1  0  Search 2  0 |
| **“stem cell research” and “clinician education”** | Search 1  Nov 13, 2020  Search 2  May 25, 2022 | Search 1  2010-2020  Search 2  2021-2022 | Search 1  article and review  Search 2  article and review | Search 1  0  Search 2  0 |

Table 3. Search strategy details for PubMed

| **Search String for PubMed** | **Search Conducted** | **Years of Search** | **Article Types** | **# of Results** |
| --- | --- | --- | --- | --- |
| **“regenerative medicine” and “medical education”** | Search 1  Nov 13, 2020  Search 2  May 25, 2022 | Search 1  2010-2020  Search 2  2021-2022 | Search 1  journal and review article  Search 2  journal and review article | Search 1  135  Search 2  74 |
| **“stem cell research” and “medical education”** | Search 1  Nov 13, 2020  Search 2  May 25, 2022 | Search 1  2010-2020  Search 2  2021-2022 | Search 1  journal and review article  Search 2  journal and review article | Search 1  30  Search 2  18 |
| **“regenerative medicine” and “physician education”** | Search 1  Nov 13, 2020  Search 2  May 25, 2022 | Search 1  2010-2020  Search 2  2021-2022 | Search 1  journal and review article  Search 2  journal and review article | Search 1  2  Search 2  2 |
| **“stem cell research” and “physician education”** | Search 1  Nov 13, 2020  Search 2  May 25, 2022 | Search 1  2010-2020  Search 2  2021-2022 | Search 1  journal and review article  Search 2  journal and review article | Search 1  0  Search 2  1 |
| **“regenerative medicine” and “clinician education”** | Search 1  Nov 13, 2020  Search 2  May 25, 2022 | Search 1  2010-2020  Search 2  2021-2022 | Search 1  journal and review article  Search 2  journal and review article | Search 1  1  Search 2  1 |
| **“stem cell research” and “clinician education”** | Search 1  Nov 13, 2020  Search 2  May 25, 2022 | Search 1  2010-2020  Search 2  2021-2022 | Search 1  journal and review article  Search 2  journal and review article | Search 1  0  Search 2  0 |
